# Supplementary material for: Synaptic vesicle undocking induces low-frequency depression
Source: eLife. 2026 Oct 12;15:RP110329. doi: 10.7554/eLife.110329 (PMC13657630; doi:10.7554/eLife.110329)
Supplement: Peer review history

# Editor report: eLife Assessment

**DOI:** 10.7554/eLife.110329.3.sa0

**Authors:** Henrique von Gersdorff

**Affiliations:** Oregon Health and Science University United States

**Keywords:** Convincing, Fundamental

This **fundamental** work substantially advances our understanding of short-term plasticity mechanisms by providing evidence for release-independent low-frequency synaptic depression that reflects a model that proposes a redistribution of vesicles within the readily releasable pool, via a reduction in docking site occupancy due to vesicle undocking. The evidence supporting this model is **convincing**, with rigorous modeling, electrophysiological data and computational analysis. The work will be of broad interest to cellular neuroscientists and synaptic physiologists.

# Referee report: Reviewer #1 (Public review)

**DOI:** 10.7554/eLife.110329.3.sa1

Summary:

In this work, the authors investigate the mechanisms of low-frequency synaptic depression at cerebellar parallel fiber to interneuron synapses using unitary recordings that allow direct quantification of synaptic vesicle release. They show that sparse stimulation can induce robust synaptic depression even in the absence of substantial vesicle consumption, and that this depressed state is rapidly reversed when stimulation frequency is increased. To account for these observations, the authors propose a model in which low-frequency depression reflects a redistribution of vesicles within the readily releasable pool, in particular a reduction in docking site occupancy due to vesicle undocking.

Strengths:

I found the experimental work to be of high quality throughout. The use of simple synapse recordings to count individual vesicle release events is particularly powerful in this context and allows questions to be addressed that are difficult to approach with more conventional approaches. The demonstration that low-frequency depression can occur independently of prior vesicle release, together with the rapid recovery observed during high-frequency stimulation, places strong constraints on possible underlying mechanisms and represents a clear strength of the study.

The modeling framework is clearly laid out and helps organize a broad set of observations across stimulation frequencies. Several of the experimental tests appear well motivated by the model, including the recovery train experiments, the analysis of failures, and the use of doublet stimulation. Taken together, the data provide a coherent phenomenological description of low-frequency depression and its relationship to vesicle availability within the readily releasable pool.

Weaknesses:

The major concerns raised during the initial review have been successfully addressed through substantial revisions of both the manuscript and the presentation of the model. The distinction between experimental observations and model-based interpretation is now considerably clearer, and the discussion more appropriately reflects the explanatory nature of the modeling framework.

# Referee report: Reviewer #2 (Public review)

**DOI:** 10.7554/eLife.110329.3.sa2

Summary:

Silva and co-workers exploit their previously established methods of analyzing release events at single parallel fiber to molecular layer interneuron synapses. They observed synaptic depression at low transmission frequencies (< 5 Hz) which rapidly recovers during high-frequency transmission. Analysis of the time course of low-frequency depression revealed an initial rapid and a slow linearly increasing time course. Strikingly, the initial depression occurred even in the absence of proceeding release arguing against vesicle depletion as the underlying mechanism.

Strengths:

The main strength of the study is the careful demonstration of an interesting synaptic phenomenon challenging the classical vesicle-centered interpretation of synaptic depression.

Weaknesses:

There are no weaknesses.

# Referee report: Reviewer #3 (Public review)

**DOI:** 10.7554/eLife.110329.3.sa3

Summary:

The manuscript builds on the observation that, at some synapses, low-frequency stimulation causes synaptic depression which can be reversed by subsequent high-frequency stimulation. Such low-frequency depression (LFD) cannot be easily explained by the depletion of a single vesicle pool. Here, Silva and colleagues propose a model of activity-dependent vesicle trafficking to explain LFD at synapses between cerebellar granule cells and molecular layer interneurons.

Strengths:

Overall, LFD is interesting and worthy of examination, and the authors provide new experimental results that are of the high quality expected from this group.

Weaknesses:

The study proposes a novel model of vesicle trafficking that is not explained by known biological mechanisms, and the manuscript does not adequately compare or discuss alternative models.

I have several concerns about how the authors interpret the data. First, the manuscript's primary conceptual advance is the idea that LFD involves vesicle undocking, rather than depletion. However, most experiments were performed under conditions that promote vesicle depletion (3 mM extracellular Ca2+). When experiments were repeated in physiological Ca2+, there appeared to be little or no LFD (stats are not provided). Second, the RS/DS/DU/undocking model, though not outside the realm of possibility, is not readily explained by known mechanisms and is only loosely supported by experimental findings. Third, when simulating LFD, the authors do not compare alternative models and use inappropriate language to imply that a model fit represents the truth (e.g. "the finding of identical experimental and simulated values confirms that the undocking mechanism accounts for LFD"). Finally, the model is presented in an overly complicated manner. The sheer amount of terms and nomenclature makes the manuscript confusing and difficult to read. Overall, the manuscript would benefit from added experiments and more statistics, a better justification and evaluation of the model, and more nuanced language.

Comments on revised version.

I appreciate the authors' detailed responses to my initial review of the manuscript. My suggestions reflect a sincere attempt to improve the clarity and accuracy of the paper. I disagree with some of the authors conclusions and their responses to my comments, but do not wish to make any further suggestions. The authors are entitled to their own views.

A final note: If the authors want to refrain from making definitive statements on underlying cellular mechanisms, they should consider amending the title of the manuscript.

# Author comment: Author response

**DOI:** 10.7554/eLife.110329.3.sa4

**Authors:** Melissa Silva, Federico F Trigo, Isabel Llano, Alain Marty

**Affiliations:** SPPIN, Université Paris Cité Paris France; Instituto de Investigaciones Biológicas Clemente Estable Avenida Italia 3318, Montevideo 11600 Uruguay; CNRS and University of Paris Paris France; Université Paris Cité Paris France

The following is the authors’ response to the original reviews.

**Public Reviews:**

**Reviewer #1 (Public review):**

Summary:

In this work, the authors investigate the mechanisms of low-frequency synaptic depression at cerebellar parallel fiber to interneuron synapses using unitary recordings that allow direct quantification of synaptic vesicle release. They show that sparse stimulation can induce robust synaptic depression even in the absence of substantial vesicle consumption, and that this depressed state is rapidly reversed when stimulation frequency is increased. To account for these observations, the authors propose a model in which low-frequency depression reflects a redistribution of vesicles within the readily releasable pool, in particular, a reduction in docking site occupancy due to vesicle undocking.

Strengths:

I found the experimental work to be of high quality throughout. The use of simple synapse recordings to count individual vesicle release events is particularly powerful in this context and allows questions to be addressed that are difficult to approach with more conventional approaches. The demonstration that low-frequency depression can occur independently of prior vesicle release, together with the rapid recovery observed during high-frequency stimulation, places strong constraints on possible underlying mechanisms and represents a clear strength of the study.

The modelling framework is clearly laid out and helps organize a broad set of observations across stimulation frequencies. Several of the experimental tests appear well-motivated by the model, including the recovery train experiments, the analysis of failures, and the use of doublet stimulation. Taken together, the data provide a coherent phenomenological description of low-frequency depression and its relationship to vesicle availability within the readily releasable pool.

We thank the Reviewer for his positive assessment of our work.

Weaknesses:

While the experimental results are strong, the manuscript would benefit from rebalancing the strength of the mechanistic conclusions drawn from the modelling in light of its limitations. The framework is clearly useful and provides a coherent interpretation of the data, but it is not uniquely constrained by the experimental observations, and alternative models or interpretations could plausibly account for the findings. The use of different model regimes concatenated across time, with substantially different parameter values, highlights the abstract nature of the approach. For these reasons, the model seems best presented as one plausible explanatory framework rather than a definitive biological mechanism. Clarifying the distinction between data-driven observations and model-based inferences would help readers assess which conclusions are strongly supported and which remain more speculative.

The interpretation of the Ca2+-related experiments would benefit from more cautious wording. The absence of detectable changes in presynaptic Ca2+ signals does not exclude more localized or subtle Ca2+-dependent mechanisms, and conclusions regarding Ca2+ independence should therefore be framed accordingly. In addition, while low-frequency depression is still observed at reduced extracellular Ca2+, these experiments appear less diagnostic of the specific model-derived mechanism emphasized elsewhere in the manuscript - namely, a selective reduction in docking-site occupancy - and should be discussed with appropriate qualification in the text.

Concerning Ca2+ signals, the Reviewer is right. While we found no change in Ca2+ signalling apart from a slow Ca2+ accumulation during long trains at 1 Hz, the possibility of an undetected change cannot be excluded. We have added a word of caution in this direction on p. 11. Concerning the 1.5 mM Ca2+ experiments, the Reviewer presumably alludes to the first recovery train (yellow) point in Supplementary Fig. 2C. This is also the last point (s11) of the slow train at 0.5 Hz because no delay at all was interposed between the slow train and the recovery train. We have now included one more experiment (with a present total number n = 6), and we have corrected Fig. S2C accordingly. In the new version the depression measured for s4-s10 vs s1 during the 0.5 Hz trains is 0.69 +/- 0.05 (p = 0.00058, paired one-tail t-test). The ratio of the s1 value of the recovery train compared to control s1 is 0.83 +/- 0.08 (p = 0.028, paired one-tail t-test).

Major points:

(1) Clarify and qualify mechanistic claims derived from the model.

Throughout the manuscript, changes in model parameters are at times described as if they directly reflected underlying physiological mechanisms. As a result, the conceptual distinction between experimentally observed phenomena, model-derived variables, and biological interpretation is not always clear. Several conclusions in the Results and Discussion are phrased as mechanistic statements, although they rest on assumptions intrinsic to the modelling framework. The authors should systematically review the text and explicitly distinguish between (i) experimentally observed changes in synaptic responses and (ii) inferences about vesicle docking states or transitions within the model.

In particular, statements implying that vesicle undocking is the mechanism underlying low-frequency depression should be rephrased to reflect that this is an interpretation within the proposed framework rather than a uniquely demonstrated biological process. For example, statements such as "Low-frequency depression is caused by synaptic vesicle undocking" should be replaced with formulations such as "Within the framework of our model, low-frequency depression is accounted for by a redistribution of synaptic vesicles away from docking sites" or "Our results are consistent with a model in which changes in vesicle docking-state occupancy contribute to low-frequency depression."

A particularly problematic example is the statement that "these experiments further confirm that LFD only involves a decrease in δ, without accompanying changes in ρ or IP size." Here, an experimentally defined phenomenon (LFD) is directly equated with changes in model-derived variables. Such statements should be revised to make clear that δ, ρ, and IP size are inferred quantities within the model, and that the experimental data are interpreted through this framework rather than directly confirming changes in these parameters. Similarly, overgeneralizing statements such as "Undocking therefore represents the key mechanism controlling short-term depression across stimulation frequencies" should be softened to reflect that this conclusion emerges from the model rather than from direct experimental evidence.

As suggested, we clarify the distinction in the revised version between experimental data and modelling, and we refrain from making definitive statements on underlying cellular mechanisms.

(2) Address the biological interpretation of time-dependent model regimes.

The model relies on distinct parameter regimes applied at different time points, with some transitions effectively suppressed in certain regimes. While this approach captures the data well, its biological interpretation remains unclear. The authors should either (i) expand the discussion to outline plausible biological processes that could give rise to such regime changes (for example, calcium-dependent modulation of transition rates or activity-dependent changes in vesicle state stability), or (ii) more explicitly frame this aspect of the model as a descriptive abstraction rather than a mechanistic proposal. This further underscores the need to clearly separate the descriptive role of the model from claims about underlying biological mechanisms.

We thank the Reviewer for drawing our attention to this important point. Below 10 ms, rate constants are largely determined by the large-amplitude, fast-decaying Ca2+ signal occurring near voltage-dependent Ca2+ channels (‘Ca2+ nanodomain’). After 10 ms, the rate constants depend on the low-amplitude, slowly decaying Ca2+ signals averaged over the entire varicosity (‘volume-averaged Ca2+’). We explain this better in the revised version (Materials and Methods, p. 21).

(3) Reframe conclusions drawn from calcium-related experiments.

The calcium imaging data demonstrate no detectable changes in the measured presynaptic calcium signals under the tested conditions, but they do not rule out that calcium signals contribute in ways undetectable by the assay. Conclusions should therefore be revised to reflect this limitation, avoiding statements that exclude a role for calcium-dependent mechanisms. Wording such as "we did not detect evidence for..." would be more appropriate than conclusions implying the absence of an effect.

Similarly, while low-frequency depression is still observed at reduced extracellular calcium (1.5 mM Ca^2+^), the specific mechanistic signature emphasized elsewhere in the manuscript - namely a selectively reduced first response during a high-frequency recovery train - is no longer apparent. These experiments should therefore be discussed as consistent with the proposed framework, but not as providing independent support for a selective reduction in docking-site occupancy. Explicitly acknowledging this limitation would improve clarity and avoid overinterpreting these data.

This has been discussed above (‘weaknesses’).

(4) Soften interpretations based on non-significant comparisons.

In several places, comparisons that do not reach statistical significance are used to argue for equivalence between conditions (for example, comparisons involving failure versus non-failure trials or different LFD conditions). These conclusions should be revised to emphasize the limits of statistical power and framed as a lack of evidence for a difference rather than evidence of independence.

We have amended this point in the revised version.

**Reviewer #2 (Public review):**

Summary:

Silva and co-workers exploit their previously established methods of analyzing release events at single parallel fiber to molecular layer interneuron synapses. They observed synaptic depression at low transmission frequencies (< 5 Hz), which rapidly recovers during high-frequency transmission. Analysis of the time course of low-frequency depression revealed an initial rapid and a slow linearly increasing time course. Strikingly, the initial depression occurred even in the absence of preceding release, arguing against vesicle depletion as the underlying mechanism.

Strengths:

The main strength of the study is the careful demonstration of an interesting synaptic phenomenon challenging the classical vesicle-centered interpretation of synaptic depression.

We thank the Reviewer for his positive assessment of our work.

Weaknesses:

No major weaknesses were identified by this reviewer.

The finding of release-independent synaptic depression is important and would have widespread implications. Therefore, some more analyses to increase the confidence in these findings could be performed.

My concern is whether rundown could explain the findings. If the rate of failures in s1 increases and at the same time the amplitude decreases during the experiments, an apparent depression in s2 could arise. The Supplementary Figure 5A addresses run-down, but the figure is not easy to understand, and, as far as I understood, it does not address the question of whether the release-independent depression could be caused by a rundown. To address this, the analysis of Figure 5 could be repeated by investigating the failure rate and amplitude separately or by analyzing the 1st and 2nd half of the recordings separately.

The Reviewer makes a very important point that had escaped our attention. If the responses were declining over the course of an experiment, near the end of the recordings, a high proportion of failures would be associated with a weak response to the second AP. This could distort the relation between initial failures and amount of LFD, perhaps to the point of indicating LFD after failures when there were none. As suggested by the Reviewer, we tested this possibility by examining the stability of the synaptic responses during experiments. We found a mean s_1_ value of 0.87 ± 0.13 for the first half of the experiments used in Fig. 5, and of 1.10 ± 0.17 for the second half (p > 0.05, n = 10). This analysis shows that there was no rundown during these experiments. We show in Author response image 1 a plot of s1 as a function of train number in these experiments, for two examples as well as for the average across frequencies and across experiments. These plots do not suggest any artefactual correlation between failures, mean s1, and rundown.

**Author response image 1.** Plot of s1 as a function of train number for the experiments of Fig. 5

In response to a request of Reviewer 2, Author response image 1 illustrates the evolution of s1 values as a function of train number for the experiments used to produce Figure 5. In each experiment, about 20 s1 values were obtained at two ISIs (either 10 ms and 500 ms, or 800 ms and 1600 ms). Author response image 1 shows two examples of s1 values as a function of train number (these values fluctuate widely between 0 and 3), and the average across cells and ISI values. There is no indication of a rundown of s1 values as a function of train number.

**Reviewer #3 (Public review):**

Summary:

The manuscript builds on the observation that, at some synapses, low-frequency stimulation causes synaptic depression, which can be reversed by subsequent high-frequency stimulation. Such low-frequency depression (LFD) cannot be easily explained by the depletion of a single vesicle pool. Here, Silva and colleagues propose a model of activity-dependent vesicle trafficking to explain LFD at synapses between cerebellar granule cells and molecular layer interneurons.

Strengths:

Overall, LFD is interesting and worthy of examination, and the authors provide new experimental results that are of the high quality expected from this group.

Weaknesses:

The study proposes a novel model of vesicle trafficking that is not explained by known biological mechanisms, and the manuscript does not adequately compare or discuss alternative models.

I have several concerns about how the authors interpret the data. First, the manuscript's primary conceptual advance is the idea that LFD involves vesicle undocking, rather than depletion. However, most experiments were performed under conditions that promote vesicle depletion (3 mM extracellular Ca2+). When experiments were repeated in physiological Ca2+, there appeared to be little or no LFD (stats are not provided). Second, the RS/DS/DU/undocking model, though not outside the realm of possibility, is not readily explained by known mechanisms and is only loosely supported by experimental findings. Third, when simulating LFD, the authors do not compare alternative models and use inappropriate language to imply that a model fit represents the truth (e.g., "the finding of identical experimental and simulated values confirms that the undocking mechanism accounts for LFD"). Finally, the model is presented in an overly complicated manner. The sheer amount of terms and nomenclature makes the manuscript confusing and difficult to read. Overall, the manuscript would benefit from added experiments and more statistics, a better justification and evaluation of the model, and more nuanced language.

We respectfully disagree with these sweeping criticisms, as described in more detail below.

Major concerns:

(1) Most experiments were performed under conditions that exacerbate depletion

In order to attribute LFD to vesicle undocking rather than depletion, it is important to show LFD under conditions where depletion is minimal. As mentioned above, the authors only report significant LFD in elevated extracellular Ca2+. In a small number of experiments performed in more physiological Ca2+ (1.5 mM), there is no depression after a single stimulus, and it is not clear that there was statistically significant depression during a low-frequency train. Several studies cited in support of LFD share this problem:

Abrahamsson et al., (2007) recorded from Schaffer collaterals in 4 mM Ca, 3-4X physiological Ca2+.

Doussau et al., (2010) recorded from Aplysia synapses in 3X Ca compared to seawater.

Rudolph et al., (2011) is cited as an example of LFD. However, this study performed experiments at high release probability cerebellar climbing fibers, and reported depression that increased monotonically with stimulation frequency, so it does not resemble the phenomenon studied in this paper. Lin et al., (2022) also largely describe monotonic depression at the calyx.

The Reviewer suggests that LFD may only occur under non-physiological conditions, if the release probability has been increased by artificially elevating the extracellular Ca2+.

The implication is that LFD is at best a curiosity with little or no significance for brain signalling. We disagree with this point of view for several reasons.

Concerning the statement ‘In order to attribute LFD to vesicle undocking rather than depletion, it is important to show LFD under conditions where depletion is minimal’: This is the purpose of the analysis shown in Fig. 5.

The statement ‘the authors only report significant LFD in elevated extracellular Ca2+’ is inaccurate. Fig. S2C shows a clear LFD in 1.5 mM Ca2+, as acknowledged by Reviewer 1 (‘low-frequency depression is still observed at reduced extracellular Ca2+’). However, we failed to provide a p-value for the depression in the initial version of the paper (p was 0.004, n = 5 with this data set; paired t-test, one-tail). In the revised version, we document the 1.5 mM results more extensively, including the incorporation of the results of an additional experiment, and an explicit statistical analysis of the data (p = 0.00058, n = 6; paired t-test, one-tail).

Concerning the statement ‘there is no depression after a single stimulus’: We find that the onset kinetics of LFD is slower in 1.5 Ca2+ than in 3 Ca2+ (respectively 1.8 ISI and 0.51 ISI, Fig. 2C and Fig. S2C). This explains that the PPR is not significantly <1 in 1.5 Ca2+ without implying any weakening of the extent of LFD at steady state.

As explained in the manuscript (p. 5), in a previous work, we developed a method to ascribe changes in SV pools, within the RS/DS model, with specific modifications of s1, s2 and s5-s8 during test 100 Hz trains (Tran et al., 2022). This method was developed in 3 mM Ca2+ conditions, and for this reason, we performed most experiments for the present work in 3 mM Ca2+.

Chiu and Carter (2024) demonstrated LFD in neocortical synapses; they performed their study in 1.2 mM Ca2+, not in elevated Ca2+.

Rudolph et al., (2011) showed low-frequency depression not only in elevated external Ca2+, but also in 0.5 mM Ca2+. While Rudolph et al., (2011) did not make an explicit link between their observations and LFD, there is no reason to doubt that these observations are an example of LFD. They showed a biphasic depression when switching the stimulation frequency from 0.05 Hz to 2 Hz. In one of the founding papers of LFD, Doussau et al., (2010) describe a biphasic depression when switching the stimulation frequency from 0.025 Hz to 1 Hz; Fig. 1 of the two papers (Rudolph 2011 and Doussau 2010) are strikingly similar.

Lin et al., (2022) would probably not agree with the statement that the depression at the calyx is ‘largely monotonic’, as they stress the finding of quasi-constant depression between 5 and 50 Hz.

The authors note that their results differ from those of Atluri and Regehr, but do not mention that a possible reason for the difference is the increased release probability in their experiments.

In fact, we clearly listed the difference in external Ca2+ as a likely source of the discrepancy by saying ‘This discrepancy presumably stems from differences in experimental conditions (room temperature, stimulation of multiple presynaptic PFs and 2 mM external Ca^2+^ concentration in the previous work, vs. near-physiological temperature, single presynaptic stimulation and 3 mM external Ca^2+^ here)’.

The authors should provide statistics for the data obtained in 1.5 mM Ca, and discuss why LFD is increased in conditions that also elevate vesicle release probability.

See our comments above: the revised version includes the requested statistics. On p. 6 of the manuscript, we do provide an explanation for the apparent lack of LFD at 1.5 Ca2+ and 2 Hz, namely a superimposition of LFD with facilitation. At 1.5 Ca2+ and 0.5 Hz, our LFD numbers are not weaker than at 3 mM Ca2+ and 0.5 Hz of 1 Hz.

Altogether, it is correct that many LFD experiments have been carried out in high release probability synapses and/or under conditions of elevated Ca2+. However, the reasons underlying these choices are diverse (in our case, to build on the previous SV pool analysis developed in Tran et al., 2022 in 3 Ca2+ conditions) and do not imply a limitation to the phenomenon. LFD is present in physiological conditions for low-to-moderate release probability synapses (as shown in our work), and altogether, there is no reason to dismiss LFD as non-physiological.

(2) Lack of biological mechanisms supporting the model

The model is presented without compelling biological support. The evidence in support of vesicle undocking comes from experiments by the Watanabe lab, which showed fewer-than expected docked vesicles under EM when cultured synapses were stimulated immediately prior to high-pressure freezing. Kusick et al were careful to note that these vesicles may have been lost to fusion.

The Watanabe lab showed an SV deficit at docking sites at times ranging from about 100 ms to several seconds (Kusick et al., 2020, their Fig. 5E). This corresponds to the ISI values where we see paired-pulse depression. In their Summary, Kusick et al., raise the possibility of SV fusion as an alternative to undocking at the 100 ms time point. But the same issue had previously been considered in Miki et al., 2018 with other techniques (their Fig. 2d), where it was shown that the SV deficit seen in paired-pulse experiments could not be explained by fusion. This leaves undocking as the most likely explanation, at least in our preparation. We have added a new paragraph on p. 14 to clarify this point.

The putative undocking Kusick describes is immediate (< 5 ms after stimulation), and it was not shown to be Ca2+ sensitive. This manuscript describes "calcium-dependent undocking" that proceeds from 10 ms - 200 ms. Multiple studies from the Watanabe lab show that a single stimulus lowers the number of docked vesicles, and subsequently, there is a transient redocking of vesicles that can be blocked by EGTA or Syt7 knockout.

This is not an accurate description of the Kusick results or of our results. In the Kusick paper, the SV deficit seen at <5 ms after stimulation is attributed to exocytosis, not to undocking. Clearly, it is Ca2+-dependent. Our manuscript describes potential calcium-dependent undocking not during the time 10 ms- 150 ms, during which our undocking rate is assumed to be calcium-independent, but starting at 150 ms and lasting a few hundred ms thereafter.

I also question the rationale for the authors' model that 2 vesicles are coupled in series to a single release site. Previous papers from this lab cited EM studies from frog and neuromuscular that showed filamentous connections between vesicles (do these synapses show LFD?). Here, the authors primarily cite their previous models to support their arguments. I encourage them to continue searching for ultrastructural evidence for 2-vesicle-docking-units and to cite such studies.

It is important to remember that our sequential two-step model was not based on EM data, but on a series of functional data including variance-mean analysis of summed SV release numbers; covariance analysis among subsequent SV release numbers; analysis of release latencies as a function of stimulus number during an AP train; analysis of SV release numbers under conditions of very high release probability. We note that the phenomenon of Ca2+-dependent docking that we proposed based on these observations has been consistent with flash-and-freeze or zap-and-freeze results from several laboratories. Concerning potential filamentous connections between SVs and the AZ plasma membrane at a distance of several 10s of nm, this has been seen not only in frog or mice neuromuscular junctions, but also at brain synapses (ex: Siksou et al., Journal of Neuroscience 2007; Cole et al., Journal of Neuroscience 2016; Fernandez-Busnadiego, Journal of Cell Biology 2010; 2013).

(3) Comparison to other vesicle models

The authors use overly assertive language to suggest that the model proves a mechanism. "Altogether, these results indicate that the slow phase of LFD ... reflects a δ decrease without significant changes in pr, in ρ or in IP size". Simulating data does not conclusively "indicate" the underlying mechanism, but the authors could state their data can be "explained by a model where..".

Please see our response above to a similar point by Reviewer 1.

However, LFD does not require activity-dependent undocking. Instead, the phenomenon has been explained by high-release probability, paired with an activity-dependent increase in either docking or release probability (Chiu and Carter, 2024; Doussau et al., 2017). Does the new model do a better job of replicating some facet of the data? If multiple models can explain the same data, how can we determine which model is correct? The "Alternative Presynaptic Depression Mechanisms" should be expanded to discuss these issues.

We could not find statements in the Chiu and Carter paper or in the Doussau et al., paper explaining LFD ‘by high-release probability, paired with an activity-dependent increase in either docking or release probability’. As far as we can see, Chiu and Carter do not propose any specific mechanism for LFD, beyond saying that depression and facilitation must be separate. Doussau et al., (their Fig. 6) clearly frame their interpretation in a sequential two-step model. As in the preceding Miki et al., paper (which they cite extensively), they assume a rapid (a few ms), Ca-dependent transition between their ‘reluctant pool’ and their ‘fully-releasable pool’, respectively homologous to RS and DS. Thus, the Doussau et al., interpretation is close to that presented in our present work, even though significant differences exist. An important difference is that Doussau et al., did not use simple synapses, so that they did not have access to key synaptic parameters such as the number of docking sites or the release probability per docking site. Consequently, the model in Doussau et al., does not have the same level of detail as ours. The revised version explains better the differences and similarity between the models of Doussau et al., and that exposed in our work (new paragraph on p. 14).

**Recommendations for the authors:**

**Reviewing Editor Comments:**

Three reviewers have seen the paper. They agree that the evidence for low-frequency depression (LFD) is solid, but they all suggest that the data with 1.5 mM extracellular calcium should be analyzed and discussed in more detail. The finding of release-independent depression is surprising, but further data analysis to investigate whether it could be caused by run-down is required. The reviewers think the study will be more complete if the authors add N's and stats for LFD in 1.5 mM Ca. In addition, the most important change would be adding clarity in the text about the limitations and simplifications of the model. Please edit the text to clearly state that the presented model provides one possible framework to account for the data, and that other models might also be plausible, and clearly describe the limitations and simplifications of the model.

Thank you for your positive assessment of our work. As explained in more detail below, we have added the requested statistics for 1.5 mM Ca data. We have also thoroughly revised our text to separate more clearly facts and interpretation. Finally, we now explain in more detail the limitations of our model.

**Reviewer #1 (Recommendations for the authors):**

Minor points

(1) Statistical analysis and reporting.

Please justify the use of one-tailed statistical tests and consider whether two-tailed tests would be more appropriate in some cases.

In Materials and Methods (p. 17), we explain that one-tailed statistics were used when the sign of any possible deviation was predicted; otherwise two-tailed statistics were used.

Exact p-values should be reported consistently rather than using threshold notation.

We have corrected this as far as we could. Exact p-values that are still missing will be provided in the version of record.

For analyses using rank-based statistics (e.g., calcium imaging and recovery experiments), please clarify which comparisons were paired and which were unpaired, and ensure that this is clearly indicated in the Methods and figure legends.

We have now clearly indicated whether these statistics were paired or unpaired.

(2) Figure clarity and presentation.

In Figure 1, clarify whether the responses shown in panel C represent measured EPSCs or derived quantities, as this is not immediately clear from the labelling.

Thank you for spotting this, the figure has been corrected.

In Figure 2, please show the variability of control responses used for normalization.

The variability of control responses used for normalization is now clearly explained in Materials and Methods (p. 18).

In Figure 3, the use of similar color schemes for different model states and for data-model comparisons is confusing and should be revised.

Thank you for spotting this; has been corrected.

In Figures 4 and 5, consider reintroducing or clarifying schematic representations of the model states referred to in the text to aid reader comprehension.

Figure 4 had already such representations. We have now modified these representations to make them simpler and clearer. We considered adding some model to Fig. 5 but could not come to any satisfactory option.

(3) Controls and interpretation of pharmacological experiments.

For experiments involving intracellular BAPTA, it would strengthen the interpretation to either include or explicitly discuss positive controls demonstrating that the manipulation was effective under the recording conditions. Alternatively, loosen the interpretation of those data.

We have modified the text as suggested by the Reviewer.

(4) Terminology and consistency.

Ensure consistent use of terminology for vesicle states and model variables throughout the text and figures, and clarify whether different labels (e.g., RS/DS versus alternative notations) refer to equivalent states.

We have revised our manuscript as suggested.

(5) Model fit at short inter-stimulus intervals.

In Figure 3B, the model appears to capture the overall trend of facilitation but shows a noticeable deviation from the data at the shortest inter-stimulus intervals. It would be helpful to clarify whether this reflects limitations of the current parameterization, known simplifications in the model (e.g., assumptions about fast calcium-dependent processes), or variability across recordings. Briefly commenting on this mismatch would help readers interpret the strengths and limits of the model in this regime.

Because of the statistical nature of data (quantal fluctuations) and simulations (Monte Carlo trials), some differences between data and simulations are unavoidable. The low simulation point at 10 ms ISI in Fig. 3B may in addition result from an imperfect transition between the two time domains (time domains 1 and 2) of the simulation. We have added a sentence in Materials and Methods (p. 22) to explain this.

(6) Model parameterization and fitting.

Please clarify how model parameters were estimated, including the fitting procedure, cost function, and any constraints applied. It would be helpful to report confidence intervals or other measures of uncertainty for key fixed parameters, and to indicate how variable these parameters are across cells or recordings. In addition, a brief discussion of how sensitive the main conclusions are to variation in key parameters (such as release probability or transition rates) would help readers assess the robustness and identifiability of the model-derived interpretations.

In addition, it is not entirely clear how model parameters and transitions differ across the concatenated temporal regimes used in the simulations, nor which parameters are held fixed versus altered or effectively suppressed. Summarizing, for each regime, the parameter values (or ranges) and active transitions-either in a table or schematic-would greatly improve transparency and help readers assess parameter identifiability and the robustness of the model-derived conclusions.

For each group of experiments, results under control conditions (8-APs at 100 Hz) were averaged together, leading to the determination of one set of parameters (N, ⍴, δ, r, s, p^r^). Next the parameters for the second time domain were obtained by trial and error to optimize the fit for Fig. 1 (PPR) data. The parameters were constrained such the PPR recovered to 1 with a 10-second ISI. Unfortunately, the parameter domain was too complex to make an unsupervised fitting procedure practical. We have now added a paragraph on p. 22 to indicate which model parameters were heavily constrained by data and which ones were less constrained. Individual synapses were not simulated. Regarding the different time domains, the probabilities rb and sb are introduced for the second time domain and rf and sf are changed as shown in Fig. Supp. 3. The kinetic parameters are reset at the beginning of each simulated AP.

(7) Clarify the scope of the model with respect to the slow component of low-frequency depression.

The manuscript identifies a slow component of low-frequency depression that persists over long timescales. While this is an interesting observation, it is not fully clear whether this component is intended to be captured by the current model or represents an additional process outside its scope. Making this distinction explicit would help readers better grasp the scope and limitations of the model.

As stated in our manuscript, modelling the slow component of LFD falls outside the scope of the present work. The set of rate constants in Fig. S3 does not produce any slow component, and we failed to find a combination of rate parameters that would produce a slow component. This is now clearly stated in the Materials and Methods section.

**Reviewer #2 (Recommendations for the authors):**

I have only the following suggestions to further improve the manuscript.

(1) It is argued that LFD is alleviated when using doublets rather than singlets (Figure 8), but is the steady-state depression in s1 between singlets and doublets at the end of the experiment statistically different?

It is unclear what should be considered steady state here. Average s_1_ values are smaller for singlets than for doublets over the last 25 points (0.58 +/- 0.05 and 0.74 +/- 0.05, p = 0.02, unpaired t-test).

(2) I do not understand Supplementary Figure 4. Does the value of zero in this plot indicate that the stimulation does not evoke any release anymore? How can this be differentiated from rundown? Can the LFD be reversed by a high-frequency stimulation?

We have rewritten the description of this figure. We have also modified the figure labelling. Finally, we have changed the size and color of the symbol at zero delay to stress that the linear fit is constrained to include this point. Altogether we trust that these changes have clarified the presentation of these results.

(3) The findings are related to Kusick et al., (2020). However, I think that in Kusick et al., (2020) as well as in Watanabe et al., (2013; doi:10.1038/nature12809), all tested time points ranging from a few milliseconds to several seconds after a stimulus show vesicle depletion except the time point of 14 ms in Kusick et al., (2020). I think these findings are, therefore, inconsistent with the much slower observed LFD.

The increase in docked SV# at 14 ms is reported not only in Kusick et al., 2020 but also in Wu et al., 2023 as well as in Ogunmowo et al., 2025. Later, at 100 ms, the number of docked SVs drops again (Kusick et al., 2020, their Fig. 5 d-e, and Ogunmowo et al., 2025), to finally recover on a time scale of seconds (Kusick et al., 2020). These data fit with our observations if LFD is due to undocking, as we propose.

(4) Page 13, 4th paragraph:

Eshra et al., (2021) provide experimental evidence for "a Ca2+ -dependent rate of SV entry into the RRP".

The Reviewer is right: Eshra et al., actually reported a weak Ca2+-dependence of the replenishment. We have therefore eliminated the Eshra reference in this sentence (note that this paper is mentioned elsewhere in the manuscript).

**Reviewer #3 (Recommendations for the authors):**

(1) Complicated terminology: I had a hard time understanding the model due to terms like δ, ρ, IP, DS, RS, RS gate, etc. Anything the authors can do to reduce the number of acronyms and simplify their description/depiction of the model will be helpful for readers.

This resembles recommendation (4) of Reviewer 1. As stated in our response above, we have entirely revised the manuscript with these issues in mind. In addition, the abbreviation list at the onset of the manuscript should help the readers.

(2) Figure 5 may represent the strongest evidence in support of an undocking model. This is the smallest figure, and I found it harder to understand. For example, I was initially confused because the blue "fail S1" traces seemed to suggest a very large PPR value. I suggest vastly expanding the size of this figure and the associated results section.

Thank you for your interest in Fig. 5. We have explained the normalization procedure in more detail in the revised version. Also, we have modified the axis label of Fig. 5B to improve clarity. Finally, we have added a paragraph to explain the reason why the RS/DS model accounts for the results of Fig. 5.

(3) Given the similar results but differing mechanistic conclusions of Doussau et al., (2017), that study merits more discussion in the manuscript.

See our response to this point above (main comment (3) by the same Reviewer).

(4) Several studies of LFD synapses have found a role for kinase activity (Silverman-Gavrila et al., 2005; Doussau et al,. 2010). This mechanism could be discussed or experimentally tested here.

As mentioned in the Discussion section, the nature of the proteins involved in LFD, or in docking/undocking, remains uncertain. It is not surprising that broad-spectrum kinases and phosphatases affect LFD, as reported in the papers quoted by the Reviewer, but the full implications of these findings will only become clear in future studies.
